# Analysis of Male Pheromones That Accelerate Female Reproductive Organ Development

**DOI:** 10.1371/journal.pone.0016660

**Published:** 2011-02-08

**Authors:** Kelly A. Flanagan, William Webb, Lisa Stowers

**Affiliations:** Department of Cell Biology, The Scripps Research Institute, La Jolla, California, United States of America; Duke University, United States of America

## Abstract

Male odors can influence a female's reproductive physiology. In the mouse, the odor of male urine results in an early onset of female puberty. Several volatile and protein pheromones have previously been reported to each account for this bioactivity. Here we bioassay inbred BALB/cJ females to study pheromone-accelerated uterine growth, a developmental hallmark of puberty. We evaluate the response of wild-type and mutant mice lacking a specialized sensory transduction channel, TrpC2, and find TrpC2 function to be necessary for pheromone-mediated uterine growth. We analyze the relative effectiveness of pheromones previously identified to accelerate puberty through direct bioassay and find none to significantly accelerate uterine growth in BALB/cJ females. Complementary to this analysis, we have devised a strategy of partial purification of the uterine growth bioactivity from male urine and applied it to purify bioactivity from three different laboratory strains. The biochemical characteristics of the active fraction of all three strains are inconsistent with that of previously known pheromones. When directly analyzed, we are unable to detect previously known pheromones in urine fractions that generate uterine growth. Our analysis indicates that pheromones emitted by males to advance female puberty remain to be identified.

## Introduction

Specialized odor cues, such as pheromones, influence the display of stereotyped behaviors [1]. Pheromones have been characterized to generate either *signaling* activity, which upon detection rapidly initiates behavior such as aggression or fear [2], [3], or *priming* activity, which usually alters hormone levels and influences behavior over several days [4], [5]. Primer pheromones emitted by males induce female estrus [6]. Pheromone-mediated estrus induction, also known as the Whitten effect in rodents, advances both male and female reproductive fitness. It has been described in many mammalian species, including rodents, sheep, goats, cattle, swine [1], [7], and has been proposed to occur in humans [8]. Pheromones have also been implicated in promoting male acceleration of female puberty, the Vandenbergh effect [9], which is characterized by the development of reproductive organs, a hallmark of which is uterine growth, and an increase in hormone signaling [5].

The identities of the chemosensory ligands that induce estrus in most species are largely unknown. In the mouse, sexual maturation of young females is promoted by odors excreted in male mouse urine [9]. Characterization of these odors has been initiated through three independent approaches. First, direct evaluation of male urinary fractions that generate the Vandenbergh effect, indicated by first estrus or uterine growth, have found that the pheromone is hydrophilic [10], [11], relatively small [12], [13], and nonvolatile [13]–[15]. It had been observed that circulating male androgens are necessary to produce puberty-accelerating urine [9], [11], [16]. Therefore in a second approach, others applied subtractive gas chromatography-mass spectrometry (GCMS) methods to screen for compounds enriched in urine from intact males compared to castrated male urine, an androgen-negative control [17]–[19]. This approach identified several compounds including isoamylamine (IAA), isobutylamine (IBA), 3,4-dehydro-*exo*-brevicomin (DHB), 2-*sec*-butyl-4,5-dihydrothiazole (SBT), 6-hydroxy-6-methyl-3-heptanone (HMH), and α/β-farnesene [17], [19]. These ligands do not have the same physical characteristics as the puberty-accelerating urine fractions identified in the first approach. However, when subsequently tested for bioactivity, several of these volatile androgen-regulated compounds were reported as primer pheromones that accelerate female puberty [17], [18] and among these, some were additionally reported to have dual functions as signaling pheromones [20]–[22]. Subsequently, several of these molecules have been shown to be ligands that directly activate vomeronasal (VNO) and other chemosensory neurons [23], [24], which are known to detect some pheromone ligands [1]. In a third approach, two androgen regulated non-volatile compounds have been reported to accelerate puberty: major urinary proteins (Mups) [25] and a hypothetical Mup peptide formed from the six N-terminal Mup residues (EEARSM) [10], [26]. In native urine, Mups are known to bind and reduce the volatility of most of the androgen-regulated small molecule ligands [27], [28]. The biological significance of most ligands that activate mouse VNO neurons has not been elucidated [29], [30] therefore, it is striking that each of these approaches identified different compounds that similarly function to advance the onset of female puberty. Whether each of these ligands is equally effective to accelerate puberty or whether they are naturally synergistic has not been investigated.

Here we have applied an assay for pheromone-mediated uterine development to inbred BALB/cJ mice [9]–[18]. We evaluated the response of wild-type and TrpC2 mutant mice, which lack the primary sensory channel of the vomeronasal organ (VNO) [31], [32], and found the mutant animals to be unresponsive to puberty accelerating pheromones. We exposed BALB/cJ females to previously identified puberty accelerating pheromones and found all to be inactive in our assay. Finally, we fractionated urine to characterize general properties of the pheromone. We find male urine produced from genetically distinct mouse strains to each have pheromone activity in similarly prepared fractions and previously identified pheromones to be undetectable from the active fraction in all three strains. Our experiments suggest that pheromones that increase uterine mass, and thereby accelerate puberty, remain to be identified.

## Results

### Pheromone-mediated uterine weight increase in BALB/cJ females

**Figure 1:**
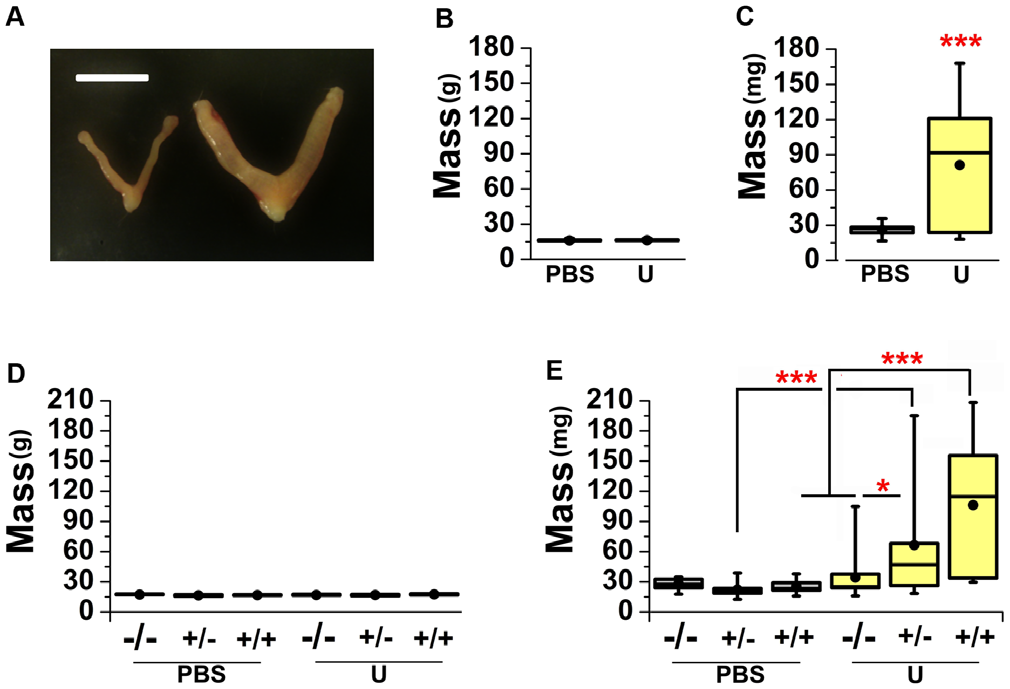
Male urinary pheromones alter female reproductive physiology. (**A**) Six-day exposure to male urine increases uterine mass, an indirect assay for puberty acceleration. Left; dissected uterus from a PBS-exposed female, right; dissected uterus from a female exposed to male urine. Scale bar, 1 cm. (**B**) Urine pheromones do not increase total body mass (t-test, p = 0.441). n = 13 per group. (**C**) Exposure to male urine results in an increase in uterine mass (t-test). n = 13 per group. (**D**) Urine pheromones and TrpC2 genotype have no significant effect on body mass (p = .132). n = 9–16 per group. (**E**) Exposure of TrpC2-/- females to male urine does not result in an increase in uterine mass (Tukey's HSD). n = 9–16 per group. PBS; phosphate buffered saline, U; male urine. (see Methods for explanation of box and whisker plot representation). *p<0.05, ***p<0.001.

To investigate the olfactory ligands that result in puberty acceleration, we utilized an *in vivo* bioassay with inbred, BALB/cJ, female mice. After weaning, chemosensory stimuli were directly applied to the nares of female juveniles twice daily for six days (see Materials and Methods for details). On day seven, both uterine and body masses were measured. Consistent with the Vandenbergh effect [5], [9], male urine promotes a significant development of the uterus of BALB/cJ females as indicated by increased wet uterine mass (Fig. 1A, C) without effect on total body mass (Fig. 1B). C57BL/6J females failed to demonstrate uterine weight increase under similar conditions (data not shown), a result which underscores the known influence of genetic background on pheromone-mediated behaviors [33]. The puberty acceleration bioassay in the BALB/cJ strain provides a screen by which putative pheromones and chemosensory neurons may be investigated.

The molecular profile of the puberty accelerating chemosensory neurons has not been identified, but surgical ablations of either the VNO or the main olfactory system have indicated that puberty accelerating pheromones are detected by unknown neurons within the VNO [34]–[36]. The function of TrpC2, the primary sensory channel of VNO neurons, is required to detect and respond to some pheromones [32], [37]–[39]. In order to analyze the role of TrpC2 function in pheromone-mediated uterine weight gain, we fully introduced the TrpC2 mutation [32] into the BALB/cJ genetic background (see Materials and Methods). Littermate progeny of *TrpC2*+/- (BALB/cJ) breeders were exposed to urine and their uterine weight was assayed blind of genotype (Fig. 1D, E). Subsequent genotyping revealed that TrpC2 null congenic females failed to increase uterine mass in response to the pheromone(s) in male urine (Fig. 1D, E), indicating that TrpC2 function is necessary to detect and respond to pheromones that increase uterine weight.

### Evaluation of pheromones previously identified to accelerate puberty

**Figure 2:**
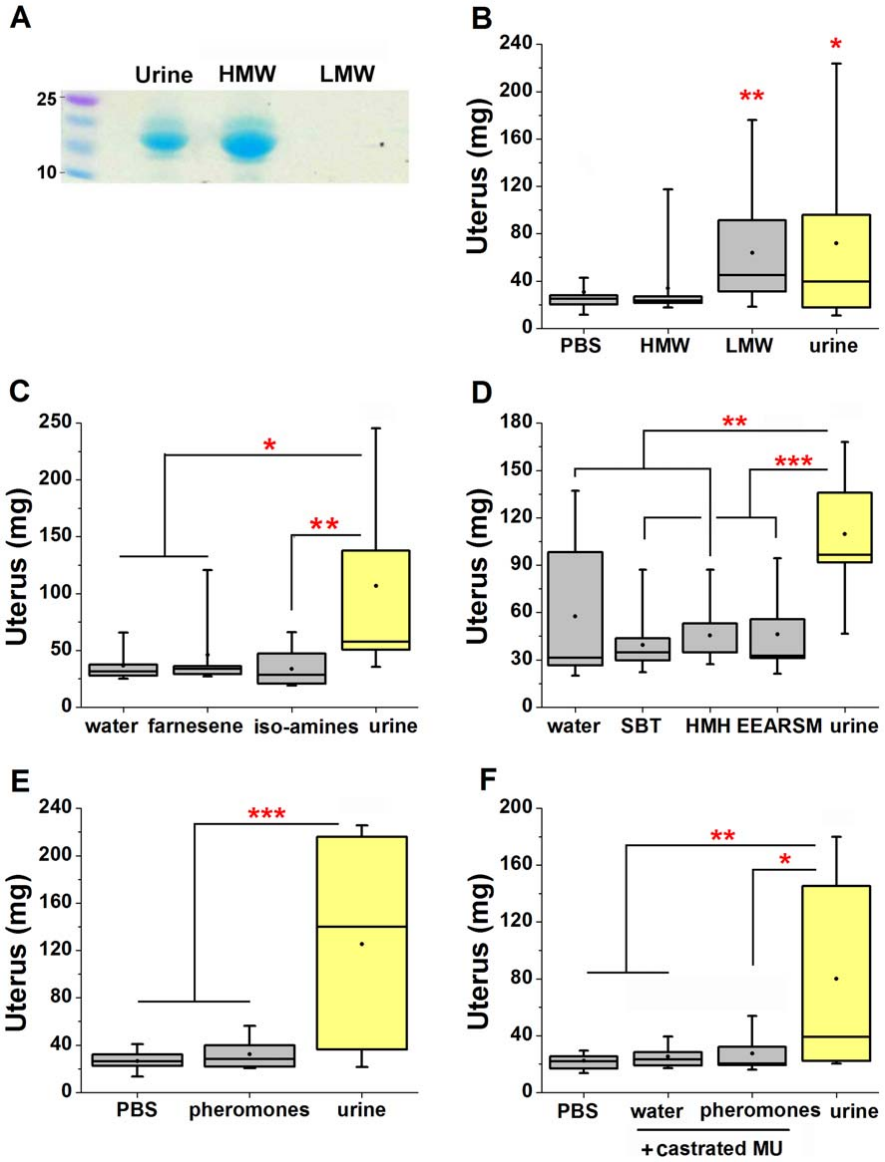
Analysis of pheromones previously identified to accelerate puberty. (**A**) SDS-PAGE of fractionation of urine by molecular mass; high molecular weight retentate (HMW >3 kDa,), low molecular weight (LMW<3 kDa). kDa size marker on left. (**B**) Exposure to LMW urine fractions increases uterine mass (Dunnett's). (End body masses are not significantly different, p = 0.237). Averages of body mass (g): PBS, 16.78; HMW, 16.46; LMW, 16.64; urine, 16.8. n = 10–19 per group. (**C**) β-farnesene (250ppm) and isoamyl- and isobutylamine (50 mM each) presented in water do not increase uterine weight (Tukey's HSD). (End body masses are not significantly different, p = 0.348). Averages of body mass (g): water, 17.43; β-farnesene, 17.71; iso-amines, 17.41; urine, 18.03. n = 7–8 per group. (**D**) SBT (2.6 ppm), HMH (2000 ppm), and EEARSM (0.5 mg/ml) presented in water or PBS do not significantly increase uterine mass (Tukey's HSD). (End body masses are not significantly different, p = 0.808). Averages of body mass (g): water, 17.25; SBT, 17.56; HMH, 17.26; EEARSM, 17.24; urine, 17.62. n = 10 per group. (**E**) A blend of known pheromones (β-farnesene, IAA, IBA, HMH, SBT, and EEARSM) in PBS does not significantly increase uterine mass (Tukey's HSD). (End body masses are not significantly different, p = 0.20). Averages of body mass (g): PBS, 16.79; pheromones, 17.05; urine, 17.36. n = 13 per group. (**F**) Neither water nor a blend of known pheromones (β-farnesene, HMH, SBT, and EEARSM) added to urine from castrated males (+ castrated MU) generates an increase in uterine mass (Tukey's HSD). (End body masses are not significantly different, p = 0.286). Averages of body mass (g): PBS, 16.81; castrate MU, 16.97; blend castrate MU, 17.03; urine, 17.45. n = 8–10 per group. *p<0.05, **p<0.01, ***p<0.001.

We next evaluated the bioactivity of pheromones that were previously reported to accelerate puberty: IAA, IBA, HMH, SBT, β-farnesene, EEARSM, and Mups. We first directly investigated the activity of the Mups. Male urine was ultrafiltrated to size fractionate the larger Mup proteins from small molecules. SDS-PAGE analysis revealed that the high molecular weight (HMW) retentate contained detectable proteins (Fig. 2A), which were the expected size of Mups [40]. When analyzed in the bioassay, we found the low molecular weight (LMW) filtrate and whole urine to each increase uterine mass, while the Mup-containing HMW retentate showed no significant bioactivity (Fig. 2B). This finding is consistent with a previous study evaluating the puberty accelerating effect of a recombinant Mup [18]. The absence of protein from LMW fractions and the lack of bioactivity in the HMW fraction argue against the role of Mups as pheromones that accelerate reproductive organ development. All of the remaining previously identified pheromones are smaller than 3000Da and are expected to be present in LMW urinary fractions.

We used three separate approaches to directly evaluate the extent to which other previously identified puberty inducing pheromones increase uterine mass. In all of our experiments, we analyzed the ligands using the concentrations and vehicles previously reported for bioactivity [10], [17], [18], [41]. First, we assessed the sufficiency of each pheromone diluted in water as previously described. Exposure of BALB/cJ females to commercially available synthetic pheromones, β-farnesene, and blended IAA and IBA, failed to significantly increase uterine mass (Fig. 2C). Custom synthesized pheromones, HMH, SBT, and hexapeptide EEARSM also lacked the ability to significantly increase uterine weight when individually tested (Fig. 2D).

Second, while these pheromones were not reported to function as an obligate blend, some invertebrates respond to blends of structurally related sex pheromones with potentiated bioactivity [42], [43]. We therefore evaluated whether the bioactivity of these previously identified pheromones was more apparent as a blend. Mice were exposed to a mixture of IAA, IBA, β-farnesene, SBT, HMH and EEARSM. This complex blend of pheromones failed to increase uterine mass (Fig. 2E). Lastly, SBT has been reported to additionally act as a signaling pheromone, but only when added to urine from castrated males (which alone does not generate bioactivity) [22]. Therefore, we aimed to determine if the previously identified pheromones accelerate puberty in conjunction with other unidentified male odors that may be present in castrated urine. In the first two approaches we observed aversion to IAA and IBA and considering the toxicity of inhaled aliphatic amines in mice [44], these ligands were excluded from this final direct assessment. As previously described [22], we added β-farnesene, SBT, HMH, and EEARSM into urine from castrated males, providing urinary context. However, we found both the urine from castrated males alone and castrated urine supplemented with the blend of pheromones similarly unable to significantly increase uterine weight (Fig. 2F). In total, all three methods of direct analyses of previously identified pheromones did not accelerate BALB/cJ reproductive organ development in our bioassay.

### Biochemical characteristics of pheromones that increase uterine mass

**Figure 3:**
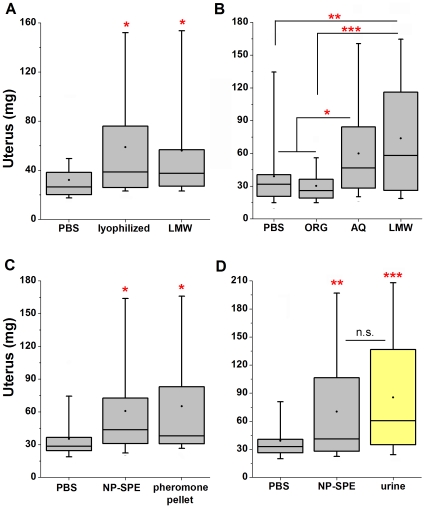
Fractionated bioactivity has biophysical characteristics that are inconsistent with known pheromones. (**A**) Lyophilization of LMW urine, to remove volatile urinary constituents, retains the bioactivity of a fresh LMW fraction (Dunnett's). (End body masses are not significantly different, p = 0.296). Averages of body mass (g): PBS, 17.90; lyoph LMW, 17.83; LMW, 17.50. n = 21–23 per group. (**B**) Pheromones that increase uterine mass are not extracted into chloroform (ORG), they are retained in the aqueous phase (AQ) (Tukey's HSD). (End body masses are not significantly different, p = 0.313). Averages of body mass (g): PBS, 17.41; ORG, 17.29; AQ, 16.98; LMW, 17.29. n = 20–35 per group. (**C**) Analysis of uterine weight folowing purification of the partially purified “pellet” by an NH_2_ column using aqueous normal phase-solid phase chromotography (NP-SPE) (Dunnett's). (End body masses are not significantly different, p = 0.786). Averages of body mass (g): PBS, 17.46; NP-SPE, 17.58; pellet, 17.62. n = 24–28 per group. (**D**) Comparison of retained bioactivity in NP-PSE fraction to total urine (Tukey's HSD; indicated p values relative to PBS). (End body masses are not significantly different. P = 0.783). Averages of body mass (g): PBS, 17.52; NP-SPE, 17.41; urine, 17.50. n = 50–56 per group. PBS; phosphate buffered saline, LMW; low molecular weight urine size fraction. n.s., not significant, *p<0.05, **p<0.01, ***p<0.001.

We next evaluated the biochemical characteristics of the activity that accelerates pubertal uterine mass increase in the urine LMW fraction. First, we determined the extent to which pheromones that increase uterine mass are volatile. We lyophilized LMW urine, which is devoid of volatile-stabilizing Mups [18], [27] and found it to retain significant bioactivity (Fig. 3A), confirming that the pheromone is not highly volatile [13], [14]. We next fractionated LMW urine by solvent extraction and both the organic and aqueous phases were screened for the ability to increase uterine mass (Fig. 3B). Hydrophobic molecules extracted into the organic phase failed to demonstrate any bioactivity while the aqueous phase significantly accelerated uterine growth (Fig. 3B), confirming that the pheromone is preferentially aqueous [10], [11].

To further identify basic biochemical properties of the pheromone, we continued to use the bioassay to track the activity through several purification steps and collected the pheromone by solvent precipitation to generate a partially purified pheromone “pellet” fraction (see Materials and Methods for details). Attempts to further isolate the pheromone with routine adsorption to C18 reverse-phase solid phase extraction (RP-SPE) columns were unsuccessful. We found the hydrophilic flow-through which was collected as a batch using C18-high pressure liquid chromatography (HPLC) demonstrated significant bioactivity in contrast to hydrophobic compounds that eluted under higher solvent strength (data not shown). We therefore considered that the pheromone might be adsorbed to hydrophilic matrices using aqueous normal phase-SPE (NP-SPE) and evaluated a small panel of normal phase matrices for eluted bioactivity. We found further fractionation of the pheromone “pellet” over an amino column under aqueous normal phase conditions to recover the puberty acceleration bioactivity (Fig. 3C). The ability to isolate the pheromone by NP-SPE methods and the inability of the pheromone to bind hydrophobic C18 columns indicate that the pheromone is hydrophilic, consistent with our results using solvent extraction (Fig. 3B). Importantly, we find both the NP-SPE fraction and total urine to be similarly effective in promoting uterine weight gain (Fig. 3D). Collectively, this analysis indicates that the biochemical characteristics of ligands in the bioactive fraction (Fig. 3) are inconsistent with those of previously identified pheromones (see Table 1).

**Table 1:** Summary of biochemical properties of stimuli.

Stimulus	MUP	EEARSM	IAA	IBA	farnesene	SBT	HMH	DHB	NP-SPE
Reference	[10]	[10]	[17]	[17]	[18]	[18]	[18]	[18]	This study
<3000Da	-	✓	✓	✓	✓	✓	✓	✓	✓
Involatile	✓	✓	-	-	-	-	-	-	✓
Aqueous	✓	✓	-	-	-	-	-	-	✓
Uterine hypertrophy	-	-	-	-	-	-	-	ND	✓

Aqueous, activity recovered by organic extraction. Uterine hypertrophy, ligand accelerated uterine hypertrophy. ND, not determined.

### The bioactive urinary fraction is similarly extracted from three mouse strains

**Figure 4:**
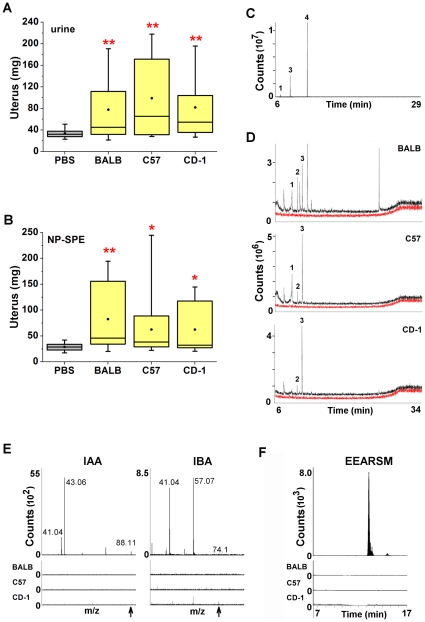
Known pheromones are not present in the bioactive NP-SPE fraction from three different strains. (**A**) Male urine of BALB/cJ, C57BL/6J, and CD-1 each diluted 1∶3 in PBS promotes uterine weight increase in female mice (Dunnett's). (End body masses are not significantly different, p = 0.839). Averages of body mass (g): PBS, 17.29; BALB/cJ, 17.47; C57, 17.40; CD-1, 17.26. n = 27 per group. (**B**) Females exposed to the NP-SPE amino-column elutions from three different mouse strains display uterine weight gain (Dunnett's). (End body masses are not significantly different p = 0.662.) Averages of body mass (g): PBS, 17.41; BALB/cJ, 17.40, C57, 17.39; CD-1, 17.11.n = 14–15 per group. (**C**) GCMS chromotograph of pheromone standards HMH (1), SBT (3), and β-farnesene (4) as indicated. Fragmentation patterns of standards were used for analysis. (**D**) GCMS total ion chromatographs for known pheromones from total urine (black trace) and bioactive NP-SPE fractions (red trace) from BALB/c, C57BL/6J, and CD-1 male donors. Detection of HMH (1), DHB (2), and SBT (3) are indicated. No extractable compounds, including these known pheromones, were detectable in NP-SPE samples. The NP-SPE red trace is offset below the urine trace to visualize the lack of detectable peaks, the y-axis is otherwise identical. (**E**) LCMS/MS analysis of bioactive NP-SPE samples from BALB/c, C57BL/6J, and CD-1 male donors does not detect the presence of IAA and IBA using fragmentation patterns obtained from standards (top) as indicated. Arrow indicates the exact mass of the parent ion. (**F**) Analysis of EEARSM peptide using the mass of the transition state during dissociation of the standard (top). EEARSM was not detectable in any of the NP-SPE samples from any strain. In panels E-F the y-axis are identical between standards and NP-SPE samples. *p<0.05, **p<0.01.

The constituents of adult male mouse urine vary significantly between strains [45]–[48] and result in strain-specific differences of male urine to stimulate chemosensory neurons [49], [50], attract females [51]–[53] and trigger ovulation [54]. To evaluate the extent to which pheromones which increase uterine mass are produced by different strains of laboratory mice, we assayed male urine from BALB/cJ, C57BL/6J, and CD-1 lineages. We found total urine from all three strains of mice to each significantly increase uterine mass (Fig. 4A). We next similarly prepared NP-SPE fractions from these individual strains and found them each to contain significant bioactivity (Fig. 4B). This suggests that pheromones which function to accelerate pubertal uterine growth emitted from BALB/cJ, C57BL/6J, and CD-1 strains share similar biochemical characteristics.

### A novel pheromone in the bioactive urinary fraction

To further evaluate the nature of our partially purified bioactivity we directly analyzed urine for the presence of previously known pheromones. The GCMS fragmentation patterns of SBT, HMH, and β–farnesene standards (Fig. 4C) as well as published fragmentation patterns of DHB [55] and α-farnesene were acquired prior to sample analyses. IAA, IBA and EEARSM standards were detected by liquid chromatography (LCMS) (Fig. 4E, F). Using these compound standards and published fragmentation data we analyzed total urine of BALB/cJ, C57BL/6J and CD-1 strains by LC- and GCMS for these previously identified pheromones. We found SBT, IAA, and DHB were readily detectable in the organic extracts of BALB/cJ, C57BL/6J and CD-1 male urine and HMH was detected in samples of BALB/cJ and C57BL/6J male urine (Fig. 4D, E). While β–farnesene, IBA, and EEARSM standards were detected by our methods we were unable to find them present in male mouse urine from any of these strains (Fig. 4C, D, E, F). Importantly, when we performed the same analysis on the bioactive NP-SPE fraction from each strain we were unable to detect any of these previously known pheromones (Fig. 4C, D, E, F). These results suggest that the pheromone enriched in the NP-SPE fraction of urine from several laboratory strains is likely to be novel.

## Discussion

Though pheromone-mediated estrus acceleration is a widespread mammalian phenomenon, the neural mechanism by which priming pheromones affect reproductive physiology and behavior is largely unknown. In order to study the chemosensory modulation of reproductive organ development, we applied a bioassay to investigate pheromone-mediated acceleration of uterine mass to BALB/cJ inbred females. We used this assay to assess the functional relevance of TrpC2 and screen for pheromones implicated in puberty acceleration. We found the function of TrpC2 to be required for the female pubertal response to male urine providing first molecular characterization of the sensory neurons that mediate this pheromone response. Our current experiments cannot identify whether the salient population of TrpC2 neurons are located in the main olfactory epithelium or in the VNO [31]. However, our results are consistent with the previously identified role of the VNO in pubertal acceleration [34]–[36] and the function of TrpC2 in signaling to pheromone-responsive neural circuits [32], [38], [39]. MHC peptides emitted by males have been reported to block the activity of estrus-inducing male pheromones thereby ensuring pregnancy in recently mated BALB/cJ females in the presence of the stud male (termed the Bruce effect) [45], [56]. Interestingly, both behavioral and electrophysiological studies find that the response to MHC ligands is similar in wild-type and TrpC2-/- mutant animals [57] indicating that the response to MHC peptides is independent of TrpC2 function. Consideration of these studies with our current findings suggests that the novel pheromone signaling that initiates first estrus during development and the MHC peptide signaling that inhibits estrus to promote pregnancy in adults utilize different signaling mechanisms.

Although we were unable to reproduce the bioactivity of previously known pheromones, the chemical properties of the bioactive urine fraction in our assay are in agreement with earlier studies which determined that the pheromone is relatively small, hydrophilic, and nonvolatile [12], [13], [15]. These properties are inconsistent with those of any previously known pheromones that were attributed with puberty accelerating activity (see Table 1). We were unable to obtain the material to use our BALB/cJ bioassay to directly evaluate the effects of α-farnesene or DHB, which have also been reported to advance puberty [18]. However, we were able to screen our partially purified “pellet” for evidence of these compounds and found no signals that matched published fragmentation data for either α-farnesene or DHB (Fig. 4C, D) [19], [41]. It should be noted that the discrepancies we observed in bioactivity of previously known pheromones may be due to differences in assay conditions, diet, housing conditions, littermate sex ratios, or the genomic variability between the outbred or hybrid experimental animals used in earlier studies and our use of the inbred, BALB/cJ, strain [10], [17], [18], [58]–[61]. Further purification of ligands that increase uterine mass will enable the identification of the detecting sensory receptors in BALB/cJ and provide the means to investigate such differences.

Our ability to purify the bioactivity using the same fractionation method (NP-SPE) from BALB/cJ, C57BL/6J, and CD-1 mice suggests that our novel bioactivity is either commonly excreted or is generated by structurally related pheromones. The hypothesis that three different strains may be producing a similar pheromone is not typical among known mouse pheromones. For example pheromones emitted from males including the female attractant, Darcin-Mup [53], the female arousing pheromone, ESP1 [38], and the pregnancy blocking MHC peptides [45] vary in expression between black (C57BL/6J) and albino (BALB/cJ) mouse strains. This expression difference of emitted ligands results in strain-specific variation of the male's ability to elicit pheromone mediated behaviors in females. Such mouse pheromones are genetically encoded and regulated by transcription. Conserved production of a pheromone with similar biophysical characteristics between three different strains may indicate that the pheromone is a metabolite produced by the common constraints of rodent physiology. The identification of the pheromone will provide insight into the evolution of priming pheromone production.

In summary, we have applied a bioassay for pheromone-mediated pubertal development in an inbred strain to characterize and identify puberty priming pheromones. We find that pheromone-mediated reproductive organ development is dependent upon TrpC2 expression and is not mediated by previously known pheromones. Future studies are required to identify the novel pheromone(s) within the NP-SPE fraction that advances the sexual maturation of female mice.

## Materials and Methods

### Animals

All procedures were approved by the Institutional Animal Care and Use Committee (IACUC) approval number 06-0298. BALB/cJ female mice were obtained from the TSRI breeding colony at 20–22 days of age and weighed. TrpC2 mutant animals [32] were backcrossed with BALB/cJ animals and the genotype was analyzed with a panel of 300 single-nucleotide polymorphisms (SNPs). This was repeated to generateTrpC2 null animals with a 99% BALB/cJ genetic background; a minimum of 3 additional backcrosses were subsequently performed. All TrpC2 mutant animals used in this study were of the BALB/cJ background. Male urine donors (CD-1, BALB/cJ, and C57BL/6J) were housed in groups of 3-5, including at least one stud male. Fresh urine from males 2–6 months of age was collected in metabolic cages and clarified by centrifugation prior to use. All male urine was from the CD-1 strain unless noted otherwise. Castrated male urine was collected from adult CD-1 males that were castrated at 3–4 weeks of age. Urine was used within 1–48 hrs of collection, kept at 4°C when stored overnight and warmed to ambient temperature prior to use. Upon weaning, female mice were individually caged in a room without males. All mice were maintained in 7.5×11×5” polysulfone cages, ventilated at 60 ACH with a 12∶12 h light/dark cycle (on at 0700 h) in a temperature-controlled room and kept on the Harlan Teklad LM-455 diet.

### Pubertal acceleration bioassay

Prior to each experiment, mice were individually housed. Animals were assigned to weight-matched group (one-way ANOVA, p>0.9). Beginning at 22–24 days of age, BALB/cJ females that weighed 12.0–15.0 g body weight, were treated twice daily with variable chemosensory stimuli painted on the external nares with a synthetic paintbrush dipped into a tube containing 0.5 ml of stimulus (except for IAA and IBA, detailed below). Male urine was collected from mice of the CD-1 strain unless otherwise indicated. To apply the stimulus, mice were restrained by hand in a supine position and slowly stroked with the stimulus until it bubbled at the nostrils, stroked again, and replaced in the cage. On average, cages were disturbed for 13 seconds and mice were handled for 6 seconds, during which ∼15 µl were applied, for each of 12 total exposures. Females were observed to be behaviorally aversive to direct application of 50 mM IAA or IBA (KAF unpublished observations) therefore; these stimuli were applied to a 0.75cm cotton ball and left in the cage as previously described [17]. On day seven, at 28–30 days of age, mice were euthanized and body weight was recorded. Animals were approximately 17 g at the end of each assay. Each uterus was rapidly dissected, removed of fat, and weighed. The progeny of TrpC2^+/-^ breeders were assayed blind of genotype and tail samples were collected for genotyping.

### Genotyping

Mice were genotyped by multiplex PCR. To identify the wild-type *TrpC2* gene, we used two primers to amplify the pore region of the ion channel: sense, 5′-GGCCATCTTCTTTTACATATGC, and antisense 5′-GAAACAAGGGAATGCAGG. The deleted gene was replaced with a PGK-neo cassette in the mutant mice [32] which was amplified using the following primers: 5′-CCTGATCGACAAGACCGGCTTC and 5′-GTGTTCCGGCTGTCAGCGCA.

### Evaluation of putative pheromones

To separate HMW from LMW urinary constituents, fresh urine was passed through Amicon Ultra-4 centrifugal filters with a cutoff of 3 kDa. Following the transfer of the LMW filtrate, the retentate was washed 2–3 times with 3–4 ml PBS and raised to original volume. Putative pheromones were dissolved as previously described. IAA and IBA (Sigma) were suspended in water at 0.05M [17]. β-farnesene (Bedoukian) was suspended in water at 250ppm (∼1.2 mM) [18], [41]. Racemic SBT [2] was suspended in water at 2.6 ppm (∼18 µM) [18]. HMH (a generous gift from Milos Novotny) was suspended in water at 2000 ppm (∼13.7 mM) [18]. The NH_2_-EEARSM hexapeptide (TSRI Peptide Synthesis Core Facility, 95% purity) was dissolved in phosphate buffered saline (PBS) at 0.5 mg/ml (∼690 µM) [10]. Pheromones were blended at these concentrations, except for isoamyl- and isobutylamine, which were provided on a cottonball inside the cage as previously described [17].

### SDS-PAGE

10 µl crude urine, HMW or LMW urinary fractions were desalted with G-50 spin columns (GE-Amersham) and loaded alongside standard ladder on a 10% acrylamide gel. Following electrophoresis, the gel was stained with Coomassie blue.

### Biochemical characterizations and fractionation procedures

To remove urinary volatiles, LMW urine was flash-frozen with liquid nitrogen, lyophilized under vacuum pressure for 24–48 hrs and stored at 4°C until use. Organic extraction of LMW urine was conducted with rigorous shaking in a glass separatory funnel using two volumes chloroform and one volume methanol. Each phase was collected and evaporated under vacuum pressure until chloroform was removed. In order to isolate the pheromone for column chromatography, LMW urine was precipitated with four volumes methanol on ice for 30 min. Following centrifugation, both fractions were dried, resuspended to original volumes, and assayed for the ability to increase uterine weight. While the precipitate had no bioactivity, the methanol solutes significantly accelerated uterine hypertrophy (p<0.001, Tukey's HSD). Thereafter, the precipitant was discarded and the supernatant was further fractionated with five volumes ice cold acetone on ice for 1 hr. Following centrifugation, each fraction was dried, resuspended to original volume, and tested in the bioassay. We found the supernatant to be inactive and acetone precipitate to significantly accelerate uterine hypertrophy (p<0.01, Tukey's HSD). Thereafter, this bioactive “pellet” was dried and stored at −80°C for up to two months. To further isolate the pheromone with NP-SPE, approximately 20 mg of material (obtained from 1 ml crude urine) were suspended in 250 µl 50% acetonitrile and applied to a 500 mg NH_2_ column (Supelco), equilibrated with acetonitrile. Several experiments were conducted to determine wash conditions that enabled us to retrieve bioactivity from the column (unpublished observations) and we found 10–15 ml 80% acetonitrile, 10% methanol to be optimal. The wash was discarded. The NP-SPE fraction was eluted with 1.0 ml water and stored at 4° up to two days. Samples were dried in a speed vacuum. All concentrated samples were reconstituted to original volume with water or PBS prior to use.

### Mass spectrometry

For GCMS, 0.5 ml chloroform was added to 0.5 ml fresh urine or the NP-SPE elution in a glass vial, vortexed, and stored overnight at −20°C. The lower organic phase was removed from thawed samples and stored at −20°C until analysis that day. 1 µl samples were analyzed by splitless injection onto a 30 m×250 um ID HP5ms column with He_2_ carrier gas at a 1.2 ml·min^−1^ flow-rate. The temperature program began at 50°C, held for 5 minutes, ramped to 300°C at 10°C·min^−1^ and held for 5 minutes. MS scans were from m/z 50–700 with a 5-minute solvent delay. For LCMS-MS, NP-SPE samples and pheromone standards were analyzed by quadrupole time-of-flight (QTOF) and a triple quadrupole mass spectrometer by single multiple reaction monitoring (SMRM). For QTOF analysis, 5 µl (∼5%) of samples were resolved online with a Sequant ZIC-HILIC column, 2.1 mm×150 mm, using a 50 mM NH_4_OAc (buffer A)/acetonitrile (ACN, buffer B) gradient (Time 0 = 10∶90, 3 = 50∶50, 5 = 50∶50, 7 = 85∶15, 10 =  off), followed by 7 min re-equilibration at 200 µl·min^−1^. The QTOF conditions included a drying gas flow rate of 9 L·min^−1^ with nebulizer pressure of 25 psi and a 100V fragmentor. The first quad was set to isolate on m/z values of 74.1 and 88.1. Collision energy was set to 15V and product ions of IAA and IBA were targeted with positive mode scans of m/z 50–200. The EEARSM peptide was analyzed by triple quad LCMS using SMRM. 5 µl samples were resolved with the Sequant ZIC-HILIC column, using a 10% ACN, 15 mM NH_4_OAc (buffer A)/90% ACN, 15 mM NH_4_OAc (buffer B) gradient (Time  = 0∶100, 2 = 0∶100, 10 = 50∶50, 15 = 75∶25, 18 =  off), followed by 5 min re-equilibration at 250 µl·min^−1^. The EEARSM 722.3 ->464.4 transition state was produced with a 250 V fragmentor and a 32V collision energy.

### Data presentation and statistical analysis

All data is graphed as box plots, providing the mean, median, and ranges of data, prior to data analysis. Whiskers span the approximated 5–95% range (usually the minima and maxima) and the box encompasses the 25–75% inner-quartile range, bisected by the median line. The mean is represented by a dot. Body weights and log_10_-transformed uterine weights [9], [18] were analyzed by a Student's t-test or by one-way ANOVA and compared pairwise using Dunnett's (using PBS as the statistical control) or Tukey's HSD (all pairwise comparisons) test as indicated.
